# The Advantages of Scientific Legal Mapping

**DOI:** 10.1017/jme.2025.47

**Published:** 2025

**Authors:** Scott Burris

**Affiliations:** Temple University, Philadelphia, United States

**Keywords:** Legal epidemiology, policy surveillance, legal research, methodology

## Abstract

Scientific legal mapping methods build on traditional comparative legal research practices, but add elements of transparency and reproducibility that can increase utility of research findings for empirical research, policymaking and public information. This comment on ”Global Legal Environment for LGBTQ+ Sexuality and Public Health” by Matthew Kavanagh et al. sets out the benefits of scientific legal research techniques and argues that legal scholars and their students can benefit from their wider adoption.

This issue includes a comprehensive comparative review of laws criminalizing LQBTQ+ sexuality by Kavanagh and colleagues.^1^ The paper exemplifies the value of comparative legal research. Encompassing 194 nations, the study allows us to see both an overall trend towards *less criminalization* but also, in a smaller number of countries, an *intensification* of criminalization. Case studies illustrate pathways to legal change in diverse national settings. A comprehensive assessment like this is indispensable as a guide to advocacy and policy reform, and bolsters the case for an international legal norm of decriminalization. These *results* are important — but this commentary focuses on the authors’ methods and argues that they should be much more widely used — and taught — in legal education and practice.

The Kavanagh study is an example of “scientific legal mapping,” sometimes also referred to as “policy surveillance methods.”^2^ Surveys of law across multiple jurisdictions are a staple of comparative health law.^3^ They typically entail legal research to obtain the text of the laws to be compared, followed by a process of categorization or coding of their relevant provisions, and usually some review steps as quality control. These methods can be highly accurate and reliable, so the move to scientific legal mapping methods is not based on a problem with the *quality* of traditional legal research. Rather, the move is driven by the aspiration to make traditional legal research more tractable for legal scholars, more accessible to our traditional policy and public audiences, and more useful for social scientists studying the health and other important effects of law.

## What Makes Legal Mapping “Scientific”?

Legal scholars all have their particular approaches to comparative legal research on a national or international scale, but there are core elements. Researchers must define the law of interest (“scoping”); they must retrieve the law to be studied (“legal research”); and they must develop an analytic framework to characterize the features of interest in the law (“coding”). These identified features are the “thing” to be analyzed by the scholar.^4^ Scholars may do the research and coding work themselves or deploy law student research assistants. They may provide a written set of instructions for the research and the coding. The findings may be captured in detailed memos for each jurisdiction, or in tables, or both. Quality control steps may be added at the end of the research process or embedded throughout in various forms of checking and double-checking, including the use of redundant research and coding. In the age of computers, there are many available tools for organizing and coding legal text, including spreadsheets, qualitative research software like DeDoose or Atlas.ti, and custom-built forms in various apps. Researchers and their RAs can easily work on shared documents in the cloud. The work is hard and requires good organization and care, but there is no sign that people in the field have concerns about the accuracy of the products this “traditional” collection of methods produces.

The move to scientific legal mapping does not require change in these methods as such, but it deploys those methods in an approach with subtly but importantly different aims. The paradigm shift to scientific legal mapping starts with the explicit goal of transforming the words of law into numeric data. It continues with the goal of creating these data in a manner that satisfies basic scientific standards of transparency and replicability. The shift to numeric legal data has very big consequences for efficiency and utility, as I will discuss; for the researcher, it requires a rethink of the analytic framework and the implementation of a procedure to consistently measure the presence, absence or other features of legal texts as numbers. Transparency and replicability do not necessarily alter the core elements of the research, but they do require that the entire process be documented, including the inclusion criteria for laws to be collected, coding conventions, and quality control. There is a growing literature on legal mapping methods^5^ and opportunities for training,^6^ both of which set out the technical innovations in the approach.

## Advantages for Legal Scholars

It may initially appear to the traditional scholar that adopting scientific legal mapping methods is a poor deal. There is nothing wrong with traditional methods, which are accepted as the state of the art in legal scholarship, while the scientific legal mapping approach requires at least some additional effort in documenting the research process and all the decisions that arise as it unfolds. Developing a coding scheme that can be applied consistently with minimal interpretation — and none of the wiggle room afforded by our beloved footnotes — takes some practice. That’s all true, but there are some benefits.

Scientific legal mapping may be easier than conventional methods in some respects. Careful documentation of inclusion criteria and coding conventions can be a time-saver, since it spares the researcher and their team the not-unfamiliar experience of trying to decide (or recall) why a law should be included or coded one way or another. These procedures also promote greater consistency within a team of researcher-coders. Turning legal text into numeric data produces a final product that is much easier to work with: legal scholars can more easily generate basic descriptive statistics and explore relationships across and within jurisdictions. The scientific mapping methods used by the Kavanagh team undoubtedly made their analysis and presentation of their findings much easier than using conventional legal research methods with textual data.

## Advantages for Comparative Research Consumers

Comparative research is useful to the world because knowing the policy landscape allows lawyers, policymakers, the media, the public and advocates to make assessments and decisions about or related to the law. Policymakers can compare their law with that of other jurisdictions and critically choose among existing legal models. The media can better contextualize legal developments. Citizens can inform themselves. Apart from lawyers, these consumers are not necessarily expert in finding law or understanding its components, so the more accessible and understandable the law is, the more value they can derive. It is therefore particularly useful that turning law into numeric data also happens to turn law into digitized information that can easily be represented in maps and charts, as we see in the Kavanagh paper. It can also be exported to the internet, where legal datasets can not only be easily shared as such, but can also be rendered as maps and tables and other visualizations; these often are designed to be interactive, allowing non-lawyers and lawyers alike to easily form queries and get understandable answers about the characteristics of laws within and across jurisdictions.^7^

Scientific legal mapping also facilitates advocacy. Kavanagh’s LGBTQ+ criminalization work, like the comprehensive mapping of HIV/AIDS policies he earlier spearheaded,^8^ fills a gap that has long hindered advocacy for human-rights approaches in health: the lack of reliable data documenting the legal status quo. For decades, advocates for enabling environments rooted in respect for human rights have done studies and made recommendations without being able to precisely identify the countries where change was most needed, or to follow the results of advocacy. In a comparable way, the CityHealth.org project of the DeBeaumont Foundation and Kaiser Permanente has used scientific legal mapping data to power a successful campaign to promote healthy policies in America’s largest cities.^9^ Starting with a set of recommended policies, the project scientifically mapped local law (originally in 40, now in 75 cities), algorithmically ranking them on an Olympic medal scale for within-policy strength and overall consistency with the recommendations. In its first five years, the proportion of cities earning overall medals doubled to 93%.^10^

## Advantages for Social Scientists

Social scientists from a range of disciplines conduct research on the effects of laws.^11^ In these studies, the law, as measured by an observable text, is nearly always the independent variable, or exposure of interest. While within social science and in the methods sections of papers, the fancy design elements (difference in differences, synthetic controls, sensitivity tests) tend to get the attention and glory, no single element is more important to the validity of an evaluation than the accurate measurement of the exposure. The well-established and growing roster of publicly available scientific legal datasets is a renewable resource that comes with the documentation researchers need to do (and publish) credible science.^12^

The use of scientific legal data in evaluation research offers opportunities for legal scholars to enhance the utility of their work. By using scientific legal mapping methods instead of traditional approaches, scholars can share, or even collaborate, with evaluation researchers to study the effects of the laws they have mapped. There is, unfortunately, also an imperative for legal scholars to improve social science. Recent research has shown that most researchers evaluating the effects of laws are not observing basic standards of transparency as to the legal research and coding, and are often simply grabbing undocumented legal information from websites that were not created with scientific use in mind.^13^ I and others in the field diagnose the problem largely as a failure of non-lawyers to appreciate the difficulty of accurate legal research and coding. Thus legal scholars have an important role to play by educating colleagues in other disciplines about legal research, and especially by serving as peer reviewers. Legal scholars may also wish to join in an effort to develop a formal reporting standard for legal research in scientific studies, and to support its adoption.^14^

## Conclusion — Resources for Legal Scholars and Their Students

Despite the advent of electronic databases and computers almost four decades ago, and the end of statute books and pocket-parts notwithstanding, the core research practices of legal scholars have not changed that much in modern times. Scientific legal mapping is perhaps the first dramatic departure from past practice we have seen in legal research. I’ve argued that its adoption can serve not only comparative legal scholars but the various consumers of research by making the product more accessible and transparent.

I close by pointing to resources that can help legal scholars and their students master the skills of scientific legal mapping. In addition to the method literature and training resources already described, there is now also software specifically designed for scientific legal mapping. Supported by the Robert Wood Johnson Foundation, my center makes access to MonQcle legal mapping free for most academic users,^15^ and CDC has its own software system called PHLIP for its legal mapping projects.^16^ These resources are well suited for teaching law students in advanced legal research or empirical research methods courses. Adopting and teaching scientific legal mapping methods is a small step for legal scholars, with a significant pay off for our field and our consumers.
